# Computed tomography myelography technique and spinal morphometry in healthy Yucatan pigs

**DOI:** 10.1371/journal.pone.0266396

**Published:** 2022-04-28

**Authors:** Lelia E. B. Barden, Lorrie Gaschen, Chiara De Caro Carella Hampton, Catherine Takawira, Chin-Chi Liu, Ali Nourbakhsh, Mandi J. Lopez

**Affiliations:** 1 Department of Veterinary Clinical Sciences, LSU School of Veterinary Medicine, Baton Rouge, LA, United States of America; 2 WellStar Atlanta Medical Center, Atlanta, GA, United States of America; USP FZEA: Universidade de Sao Paulo Faculdade de Zootecnia e Engenharia de Alimentos, BRAZIL

## Abstract

Porcine models of spinal cord injury (SCI) have an irreplaceable role in the development of experimental therapies. There is little literature regarding CT myelogram (CTM) techniques in swine and morphometry in miniature swine has not been established. A CT-guided method for performing myelography as well as reference values for spinal morphometry in healthy Yucatan miniature swine is lacking. The goal of this study is to describe a CT-guided method of performing CTM in a porcine model of SCI and to establish spinal morphometric reference values in mature Yucatan pigs. Six healthy, Yucatan sows, 9 months of age, weighing between 39–57.7kg, with no history of spinal disease, spinal injury, or neurologic deficits on physical exam were used in this study. CT myelography was performed in each sow under general anesthesia. CT scout images were used to guide needle placement at the L3-L4 intervertebral site. Once correct needle placement was confirmed using a 1ml test injection, a full dose of iodinated contrast (0.3ml/kg) was injected slowly over a 2-minute time period. Morphometry was performed using area measurements of the spinal cord (SC), vertebral body (VB), dural sac (DS), and vertebral canal (VC) at the mid-body and the intervertebral disc space of each spinal segment. Of the quantitative measurements, the spinal cord surface area had the widest range of values and the greatest coefficient of variance (CV) while those parameters for the vertebral canal had a low CV. Of the morphometric ratios, the DS:VC, had the lowest CV while the spinal cord ratios to DS and VC had the highest (>30). The vertebral canal surface area and the dural space: vertebral canal ratio may serve as reference values in future studies using this animal model.

## Introduction

Porcine models of spinal cord injury (SCI) have an irreplaceable role in the development of experimental therapies [1–6]. Contusion and transection models simulate the neuropathology and biomechanics of traumatic SCI and provide helpful information about anatomic regeneration [1]. Animal models also allow monitoring and control of the injury [1]. Porcine models have advantages in investigations of the spinal cord due to their closer anatomic resemblance to humans [3]. Imaging-guided spine procedures are mostly based on fluoroscopy, multidetector computed tomography (CT), cone-beam CT and magnetic resonance imaging (MRI) [5].

Porcine spinal neuroanatomy has been investigated in a few reports [5]. The reported porcine vertebral formula is C7, T14-15, L6-L7, S4, Cd20-23; the spinal cord in young swine extends to the sacrum, and in mature swine the it terminates in the lumbar canal [7]. The spinal cord formula is not established for all swine breeds, and it is not reported for miniature swine breeds like the Yucatan.

CT myelography (CTM) is a sensitive method used to image the spinal cord and quantify injury, swelling, or compression [8, 9]. CTM is a modification of the standard CT technique, in which the images are acquired after subarachnoid administration of non-ionic iodinated contrast medium via cisternal or lumbar puncture [10]. The goal of CTM is to obtain opacification of the subarachnoid space in order to enhance the distinction between the spinal cord, vertebral canal borders and the epidural space between the two. CTM increases delineation of compressive spinal cord pathology, enhances the identification of non-mineralized extradural and intradural extramedullary lesions and allows changes in spinal cord size to be quantified. Intraparenchymal spinal cord lesions such as ischemic myelopathy, myelitis, neoplasms, and acute non-compressive nucleus pulposus extrusions that enlarge the spinal cord are potentially detectable with CTM.

Compared to plain myelography, CTM is more sensitive for quantifying spinal components and characterizing spinal morphology in humans, horses, and dogs [11]. CTM morphometry has been established in dogs to assess the spinal cord for spinal stenosis, disc protrusion, focal attenuation of the subarachnoid space, spinal cord deformity, small spinal cord, and paraspinal muscle atrophy [11]. In dogs, common methods of CTM include cisternal puncture and lumbar puncture [12]. The thick muscling and dorsal adipose tissue layers covering the spine are a challenge for landmark palpation such as a dorsal spinal process, thus rendering the use of blind lumbar punctures difficult or impossible.

Domesticated miniature swine, specifically Yucatan swine, have major advantages over standard commercial swine for medical research models. Miniature swine share some biological characteristics with standard commercial swine, while their weight at maturity ranges from 45-100kg whereas standard swine can reach 100kg in 4 months and range from 250- 300kg at maturity [13]. The weight difference is significant, and not only reduces feed cost, but also makes them easier to house and handle [13].

There is little literature regarding CT myelogram techniques in swine. In one study, multidetector CT, cone-beam CT and MRI were compared for morphometric analysis of Landrace swine cadavers, a domestic commercial breed [14]. However, CTM techniques and morphometry in miniature swine have not been established. Important distinguishing anatomical features in porcine include a thick layer of adipose tissue and muscle over the cervical spine which makes access to the cisterna magna impractical [15]. A CT-guided method for performing myelography as well as reference values for spinal morphometry in healthy Yucatan miniature swine is lacking. Both would serve as references for miniature swine SCI patients and research models. The goal of this study is to describe a CT-guided method of performing CTM in a porcine model of SCI and to establish spinal morphometric reference values in mature Yucatan pigs.

## Materials and methods

Anesthesia and sedation were administered to all pigs to manage pain and discomfort during this study. Before initiation of the imaging study, pigs were sedated with a combination at a fixed ratio of 1.75 mg/kg of tiletamine and 1.75 mg/kg of zolazepam (Telazol^®^, Zoetis, Kalamazoo, MI) intramuscularly (IM), and once sedated, atropine was administered at a dosage of 0.04 mg/kg. Endotracheal intubation was performed after muscle relaxation was achieved via administration of isoflurane (5%; VETON®, Fluriso™, MWI, Boise, ID) in oxygen (4 L/min) via mask induction through a circle system. The same inhalational agent was then used to maintain a light plane of anesthesia in order to perform the imaging study. Cardiovascular and respiratory systems were then continuously monitored via the use of pulse oximetry, electrocardiography, capnography, and oscillometric blood pressure monitoring.

### Study population

The study was approved by the Institution for Animal Use and Care committee (Protocol number 19–080). Six healthy, Yucatan sows were used in this study. The sample size was based on an a priori power analysis using means of lumbar vertebra dimensions from existing computed tomography scans from a previous study of comparably aged Yucatan miniature swine. All were 9 months of age, weighed between 39–57.7kg, and had no history of spinal disease, spinal injury, or neurologic deficits on physical exam. The age was selected to represent a swine model of the human adolescent spine.

### Imaging protocol

Following achievement of an appropriate anesthetic plane, the sows were placed in sternal recumbency on the patient bed of the CT scanner (GE light speed, GE Hangwei Medical System, China). Hindlimbs were flexed with the pes resting on the table to help maintain a symmetrical sternal recumbent position straight and symmetrical position of the spinal column. Sows were positioned so that their spinal column was as straight with the use of foam positioning devices. Lateral and dorsal plane scout CT scans were performed from the thoracic inlet to the caudal border of the pelvis to ensure straight alignment of the pigs. A pre-contrast CT scan was then preformed from the thoracic inlet to the caudal pelvic border in a bone algorithm using 0.625 mm contiguous slices in the transverse plane and a 40cm field of view. Exposure settings for all scans were 120 kVp and 270 mAs.

**Fig 1:**
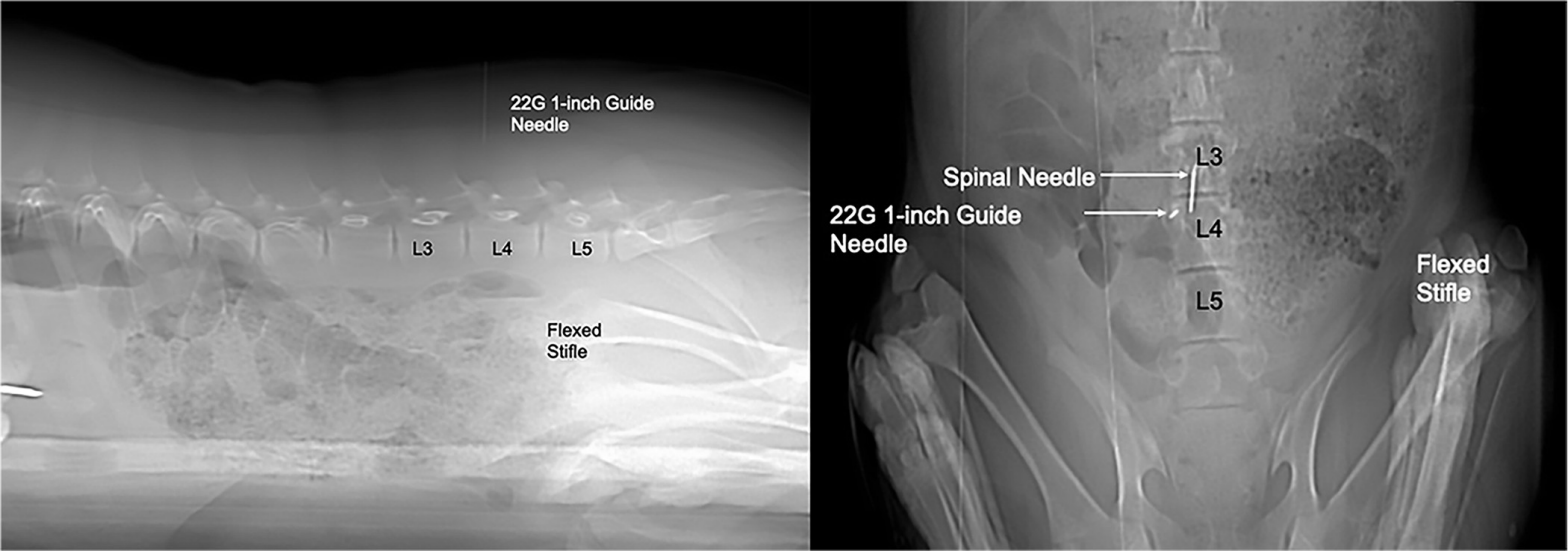
Sagittal and dorsal CT scout images showing guide needle and spinal needle placement. The guide needle is used to determine the site of insertion of the spinal needle on the skin. The needle is placed perpendicular to the long axis of the spine and immediately cranial to the border of the flexed stifle and directed perpendicularly. The spinal needle is placed into the vertebral canal immediately cranial to the guide needle and advanced until the tip meets the ventral surface of the spinal canal.

**Fig 2:**
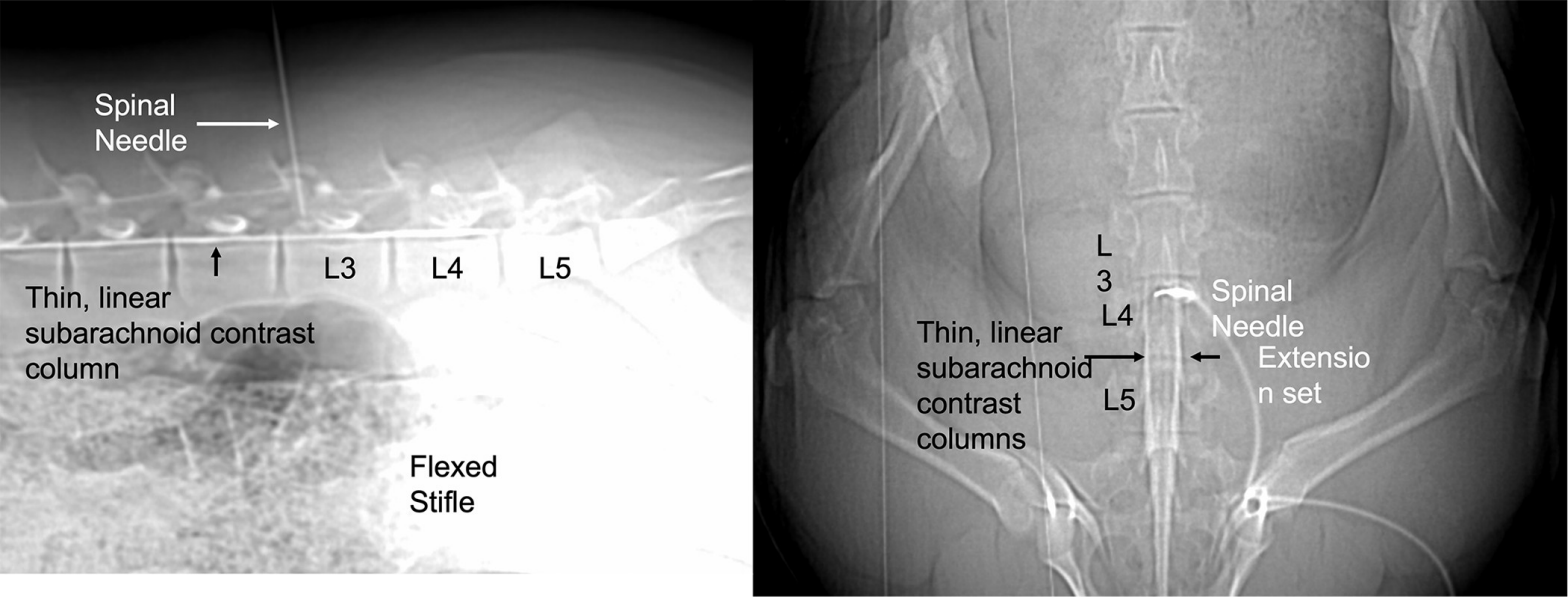
Sagittal and dorsal scout showing a test injection and linear contrast column filling of the ventral portion of the subarachnoid space. This indicates accurate needle placement so that the entire volume of contrast medium may then be injected. An epidural injection occurred in two instances and needle replacement resulted in correct injection into the subarachnoid space.

**Fig 3:**
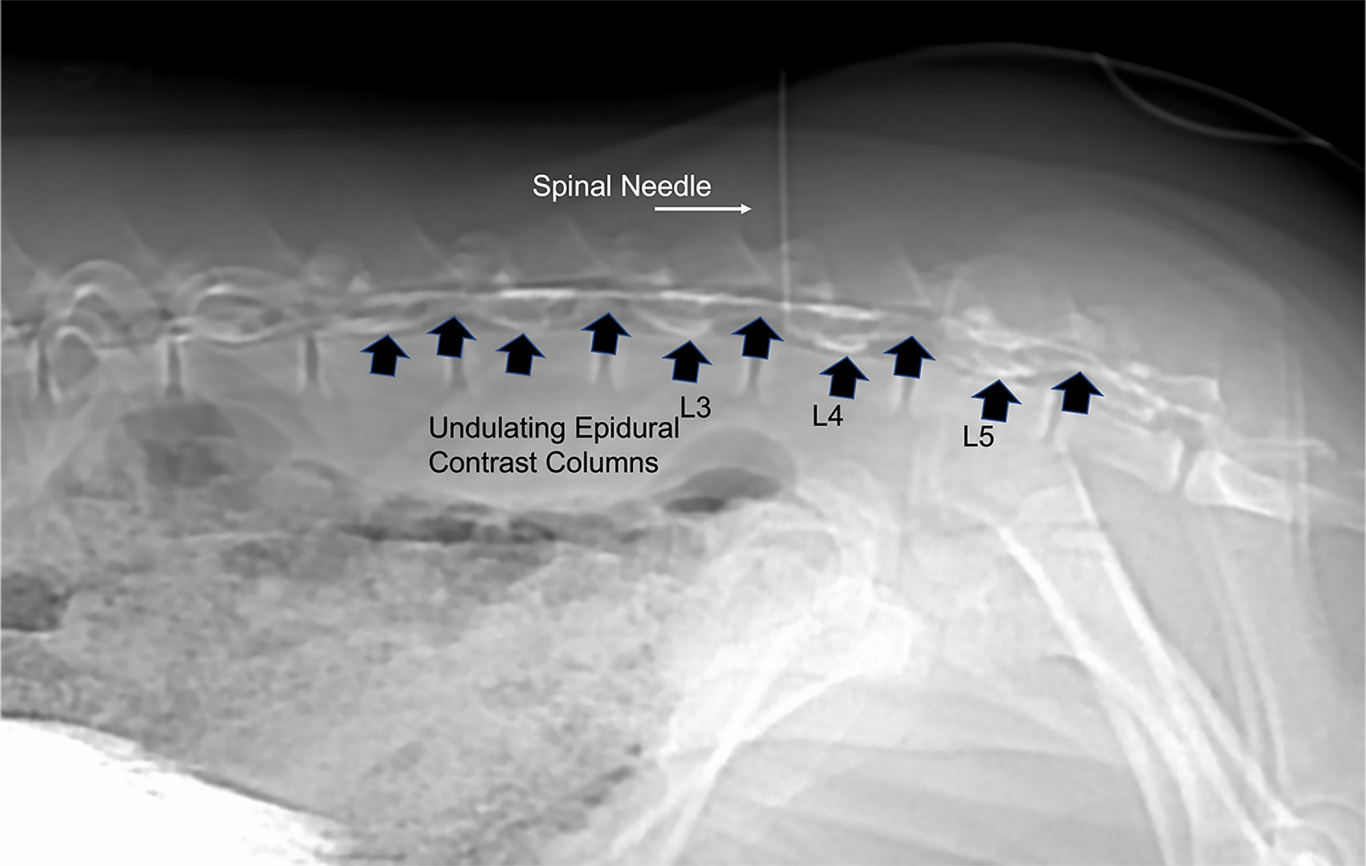
Sagittal scout showing epidural extravasation of contrast medium. This occurred in one pig and the needle was removed and a new spinal needle was placed a second time and a second test injection was performed and showed linear contrast columns of the subarachnoid space.

The lumbosacral region was aseptically prepared with alternating application of chlorhexidine (VetOne ®, MWI, Boise, ID) and 70% isopropyl alcohol scrubs. A 22-gauge, 1-inch guide needle was placed through the skin at a dorsal midline location in the approximated location of L3-L4 by using the cranial border of the flexed stifle as a landmark. The needle served as a marker for spinal needle insertion on the dorsal skin surface based off of the sagittal and dorsal scout images (Fig 1). A sterile lumbar puncture was performed with a spinal needle (20-gauge, 3.5-inch, 0.9mm x 90mm, Becton, Dickson and Company, Franklin Lakes, NJ, USA), which was advanced to the floor of the vertebral canal between L3-L4. Sagittal and dorsal scout views were used to confirm needle position (Fig 2). Subsequently, a microbore extension set (36-inch luer lock, ICU Medical, Inc., Lake Forest, Illinois) and syringe (20ml luer slip tip, Exelint® International, Co. Redondo Beach, CA) pre-filled with contrast medium were attached and used to inject contrast (Ominpaque™ Iohexol +PLUSPAK™, Marlborough, MA, USA). Initially, a test injection was performed using 1.5 mL of contrast medium into the subarachnoid space. A sagittal scout was repeated to confirm filling of the subarachnoid space which appeared as a uniform ventral linear contrast column (Fig 3). If undulating contrast filling indicated that the contrast was entering the epidural space, the needle was removed, and a new puncture and test injection performed. Once correct needle placement was confirmed, a full dose of contrast (0.3ml/kg) was injected slowly over a 2-minute time period. Immediately following contrast injection, a post contrast CT scan was repeated in an identical manner as the initial scan. Once the imaging study was completed, administration of isoflurane was discontinued, and gilts were allowed to recover from anesthesia in a quiet environment under supervision of the attending anesthetist.

### Image analysis

**Fig 4:**
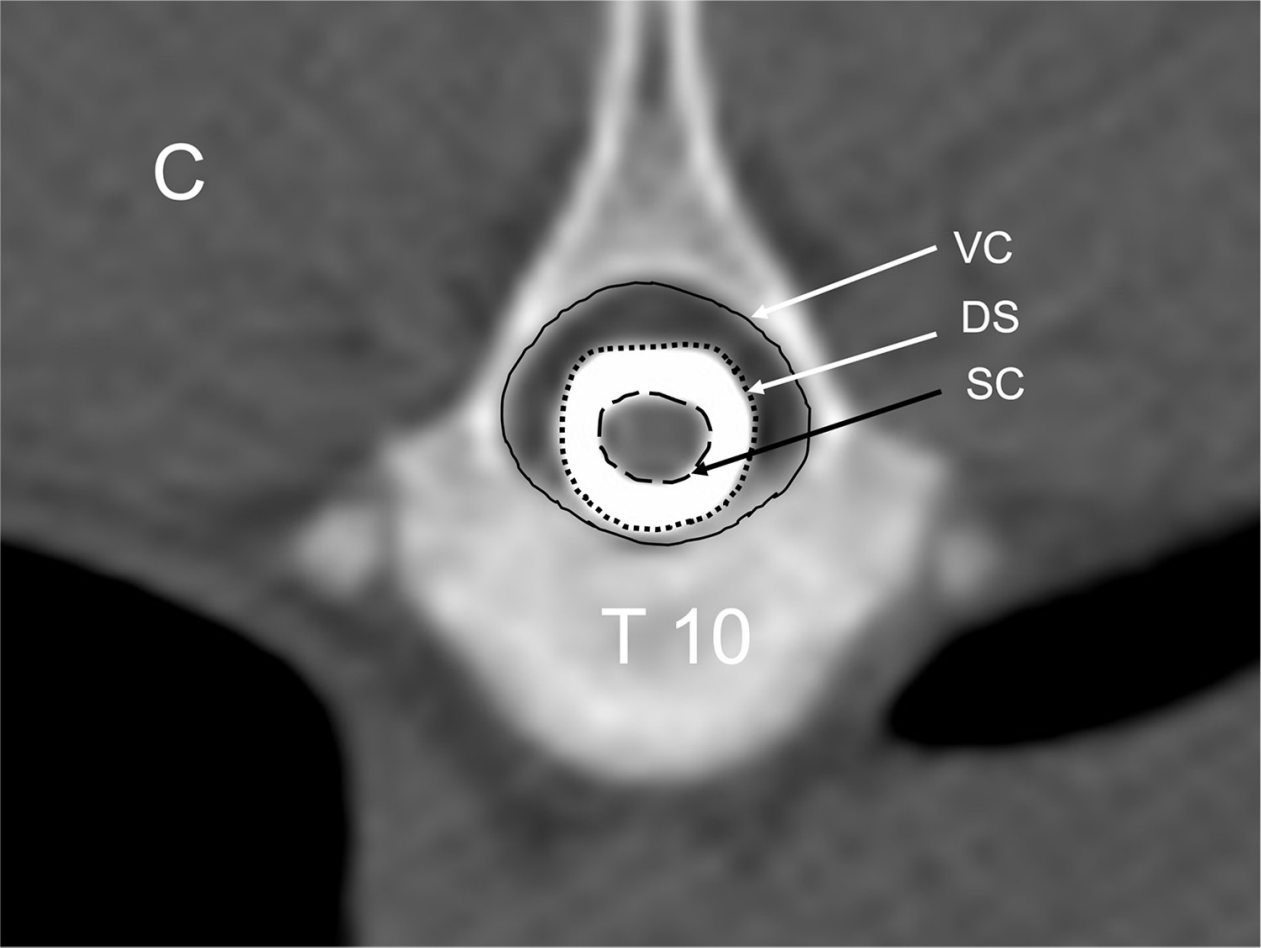
Transverse CT myelogram image showing manual delineation for area measurements. Spinal cord (SC), dural space (DS), and vertebral canal (VC). This site has the qualitative designation of C for continuous contrast filling of the subarachnoid space. C = continuous and concentric filling of the subarachnoid space.

All images were qualitatively reviewed by a board-certified radiologist (LG) using a diagnostic image viewing software (OsiriX MD v.8.5.2, Bernex, Switzerland). Transverse plane images in addition to sagittal and dorsal two-dimensional multiplanar reconstructions were reviewed in a bone window (WL: 300, WW:1500, matrix 512 x 512, 0.625 mm slice thickness). The thoracic and lumbar segments of each spine were quantitatively analyzed by the same investigator (LB) and the spinal formula recorded. Morphometric analysis of the spine was performed according to established methods [11, 14, 16, 17]. Area measurements were made of the spinal cord (SC), vertebral body (VB), dural sac (DS), and vertebral canal (VC) at the mid-body and the intervertebral disc space of each spinal segment (Fig 4). All measurements were performed by using hand-traced ROI with the image viewer’s calibrated software for area calculations three times and the average calculated. Tracings calipers were placed at the inner border of the spinal canal as shown in Fig 4. A continuous manual excursion was used to trace the maximal shape of the inner border of the canal to calculate the vertebral canal surface area. For the dural sac measurement, the caliper was placed at the outer edge of the contrast column and a manual trace of this border was completed to record the maximal surface area. The same method was used to trace the spinal cord by placing the calipers at the interface between the inner border of the dural sac filled with contrast and the outer border of the spinal cord. The total length of the spine from the cranial border of the first thoracic vertebra to the cranial border of the first sacral vertebra was measured using the sagittal multiplanar reconstruction image and the image viewer calibrated tools to normalize measurements.

**Fig 5:**
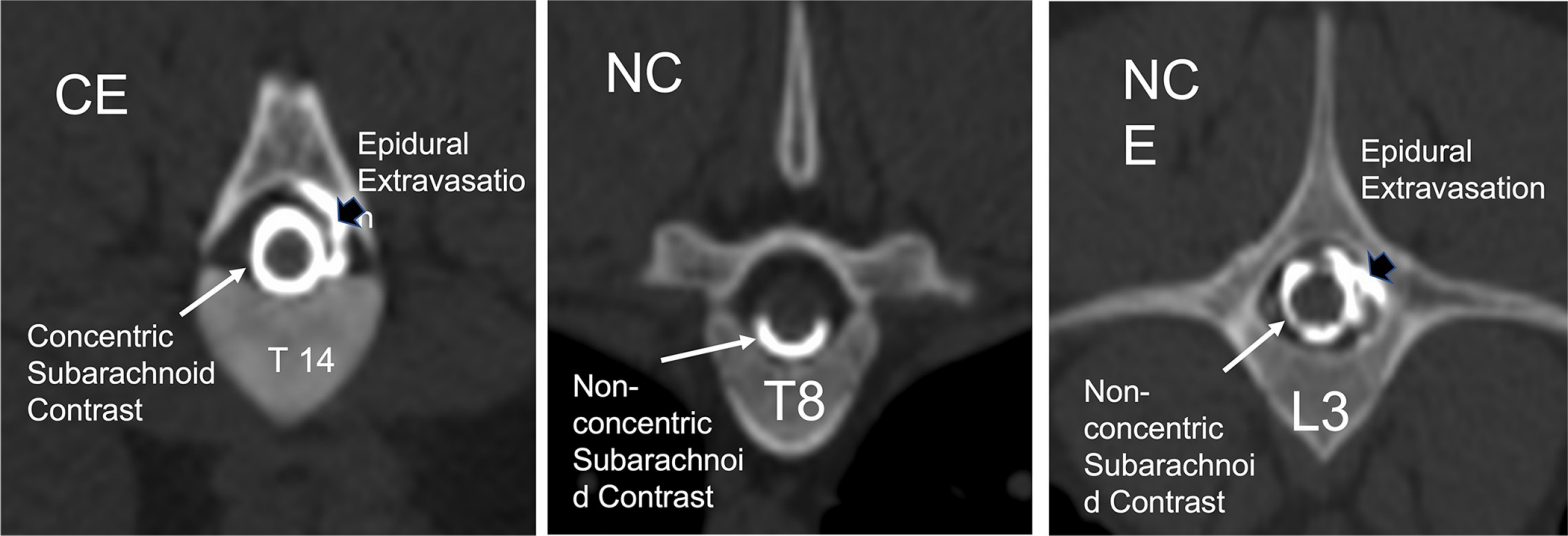
Transverse CT myelogram images showing the qualitative descriptions for contrast filling morphology. (C = concentric, NC = Non-concentric) and presence of epidural extravasation (E = epidural extravasation), or a combination of epidural contrast and non-concentric filling (NCE) or CE for concentric with epidural extravasation.

The presence of contrast filling was the criterion to determining which vertebral segments were included in area calculations. Vertebral segments with minimal contrast filling that limited visibility of the margins of the subarachnoid space, the spinal cord, and dural sac area, were not included in the study. The presence of the following CT myelographic qualitative descriptions were recorded for each vertebral segment of all sows: contrast filling morphology (C = concentric, NC = Non-concentric) and presence of epidural extravasation (E = epidural extravasation), or a combination of epidural contrast and non-concentric filling (NCE) or CE for concentric with epidural extravasation (Fig 5). Concentric was defined as complete uniform filling of the subarachnoid space with contrast medium and non-concentric as incomplete filling of the space determined from transverse images. The median, range, and interquartile range for each surface area measurement and ratio was calculated for each vertebral site in all sows. Interindividual variance was tested by the coefficient of variation (CV) and evaluated via ANOVA models with CV as the dependent, method, site as the fixed effects. When a fixed effect was detected, Tukey post-hoc comparisons were performed with least square means for the effect. Assumptions of these models (linearity, normality of residuals, and homoscedasticity of residuals) and influential data points were assessed by examining standardized residual and quantile plots using JMP Pro 15.0.0, SAS Institute Inc., Cary, NC for data analyses and Prism 8 for MacOS, GraphPad Software LLC, San Diego, CA for figures. A Shapiro-Wilk test for normality was performed for each measured parameter and ratio for each vertebral segment. The Pearson’s correlation coefficient was calculated to assess for pig weight and spinal column length compared to vertebral morphometry. Significance was set at a p<0.05.

## Results

Sows recovered from anesthesia without complications. All returned to full mobility immediately following anesthetic recovery. Table 1 includes the results of the qualitative analysis of the filling of the subarachnoid space for each site of the thoracolumbar spine included in this study. The myelograms were characterized by thin columns of contrast medium filling the subarachnoid space with minor epidural extravasation mainly in the caudal lumbar region and occasionally in the thoracic vertebral segments. The lumbar intumescence was subjectively located at L5 in 5/6 sows and not appreciated as a focal widening of the cord in one. The lumbar injection did not produce concentric filling in the cranial thoracic vertebrae T1-5 in 3/6 sows. Pooling of contrast medium by gravitational positioning was not performed but might have resulted in improved filling in that region. Filling of the cervical subarachnoid space occurred in all sows, indicating that contrast moved past the T1-5 segments cranially.

**Table 1:** Qualitative assessment of contrast filling of the subarachnoid space for each measurement at the mid vertebral body and intervertebral disc space (hyphenated). The designation of each sow, 1–6, is listed as numbers in each column for each segment.

	C	NC	CE	NCE
T1		2,4,5,6		1,3
T1-T2		2,3,4,5,6		1
T2		1,2,3,4,5,6		
T2-T3		2,3,4,5,6		1
T3		1,2,3,4,5,6		
T3-T4		2,3,4,5,6		1
T4		1,2,3,4,5,6		
T4-T5	1	2,3,4,5,6		
T5	1	2,3,4,5,6		
T5-T6		2,3,4,5,6	1	
T6	5	2,3,4,6	1	
T6-T7	5	2,3,4,6	1	
T7	1,5	2,3,4,6		
T7-T8	5	3,4,6	1	2
T8	5	2,3,4,6	1	
T8-T9	1,5	2,3,4		6
T9	4,5,6	3	1	2
T9-T10	4,5,6	3	1	2
T10	4,5,6	2,3	1	
T10-T11	4,5,6	3	1	2
T11	4,5,6	3	1	2
T11-12	4,5	3	1,6	2
T12	4,5,6	3	1	2
T12-13	4,5	3	1,6	2
T13	4,5,6	3	1	2
T13-14	1,4,5,6	3		2
T14	4,5,6	3	1	2
T14-L1	5,6	2,3	1,4	
L1	4,5,6	2	1	3
L1-L2	4,5	2	6	1,3
L2	1,4,5	2	6	3
L2-L3	1,4,5,6	2		3
L3	4,5	2	1,6	3
L3-L4	1,4,5	2	3,6	
L4	4,5		1,3,6	2
L4-L5	3,4,5	2	1,6	
L5	3,4,5	2	1,6	
L5-L6	4,		1	2
L5-S1	3,5		6	
L6	4		6	2
L6-S1	4			6

C = concentric, NC = Non-concentric, NCE = Non-concentric with epidural extravasation, CE = concentric with epidural extravasation, E = epidural extravasation.

In two of the six pigs, the initial test injection went into the epidural space. In both of these, the needle was replaced, and the second test injection resulted in correct placement and filling of the subarachnoid space. No other difficulties performing the myelography were encountered.

The vertebral formulae, location of the lumbar intumescence and conus medullaris, spinal length, body weight, and injection site for each sow are reported in Table 2.

**Table 2:** Spinal formula, body weight, spinal length, injection site, and location of the lumbar intumescence and conus medullaris of the thoracolumbar spine in 6 Yucatan sows.

Pig	Thoracic segment number	Lumbar segment number	Injection site	Lumbar intumescence	Conus medullaris	Spine length T1-S1(cm)	Body weight (kg)
**1**	**14**	**5**	**L2-L3**	**L4**	**L5-S3**	**55.5**	**57.7**
**2**	**14**	**6**	**L3-L4**	**L5**	**L6-S2**	**53.8**	**51.26**
**3**	**14**	**5**	**L3-L4**	**NA**	**NA**	**47.0**	**40.37**
**4**	**14**	**6**	**L3-L4**	**L5**	**L5-S2**	**51.8**	**42.2**
**5**	**14**	**5**	**L3-L4**	**L5**	**L5-S2**	**50.3**	**39.9**
**6**	**14**	**6**	**L3-L4**	**L5**	**L6-S2**	**51.6**	**39.0**

NA = indicates could not be identified.

**Fig 6:**
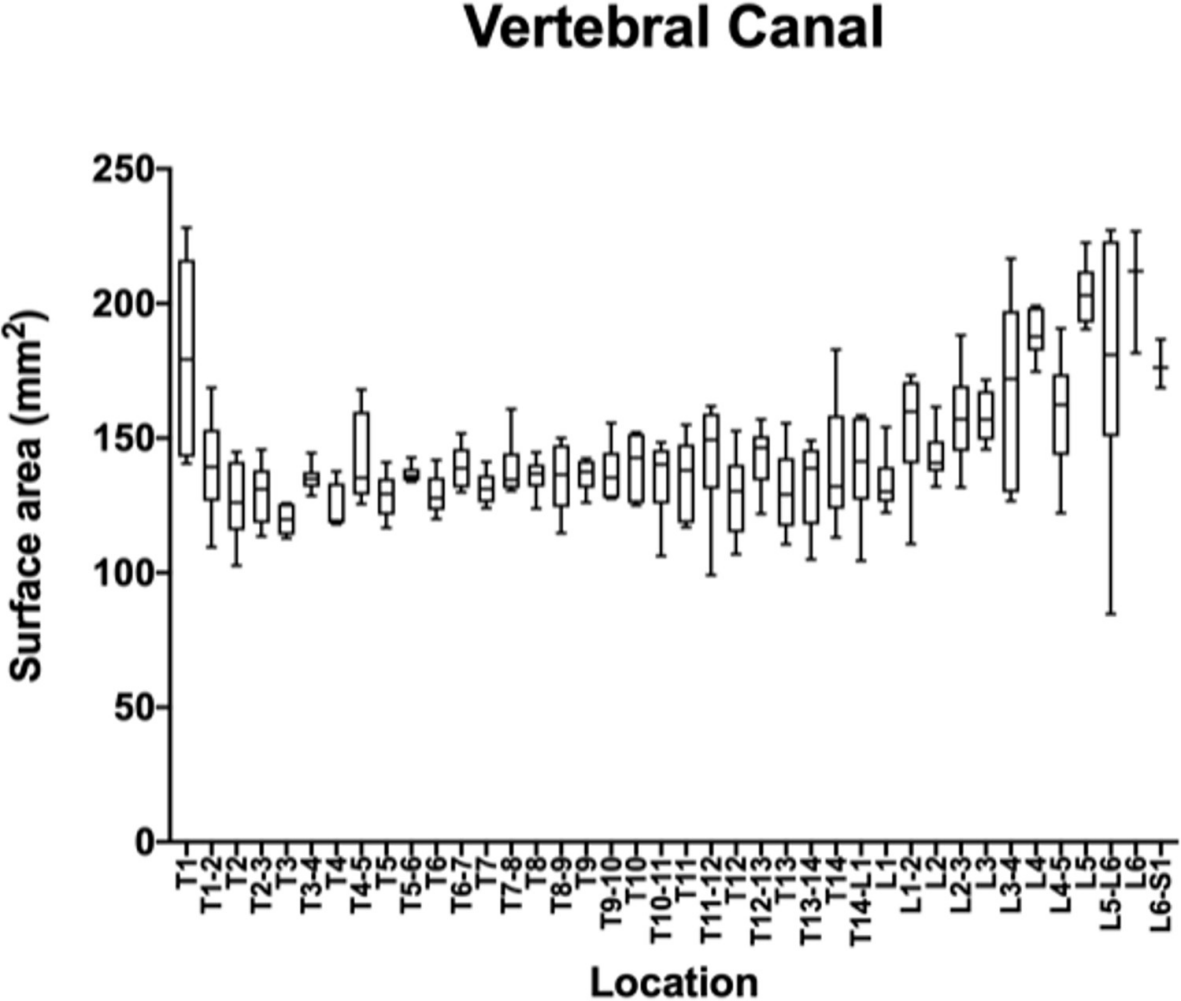
Graph of the vertebral canal surface area (mm2) in 6 Yucatan sows. The median, 75^th^, 25^th^ percentile and the range are shown for each thoracolumbar vertebral segment.

**Fig 7:**
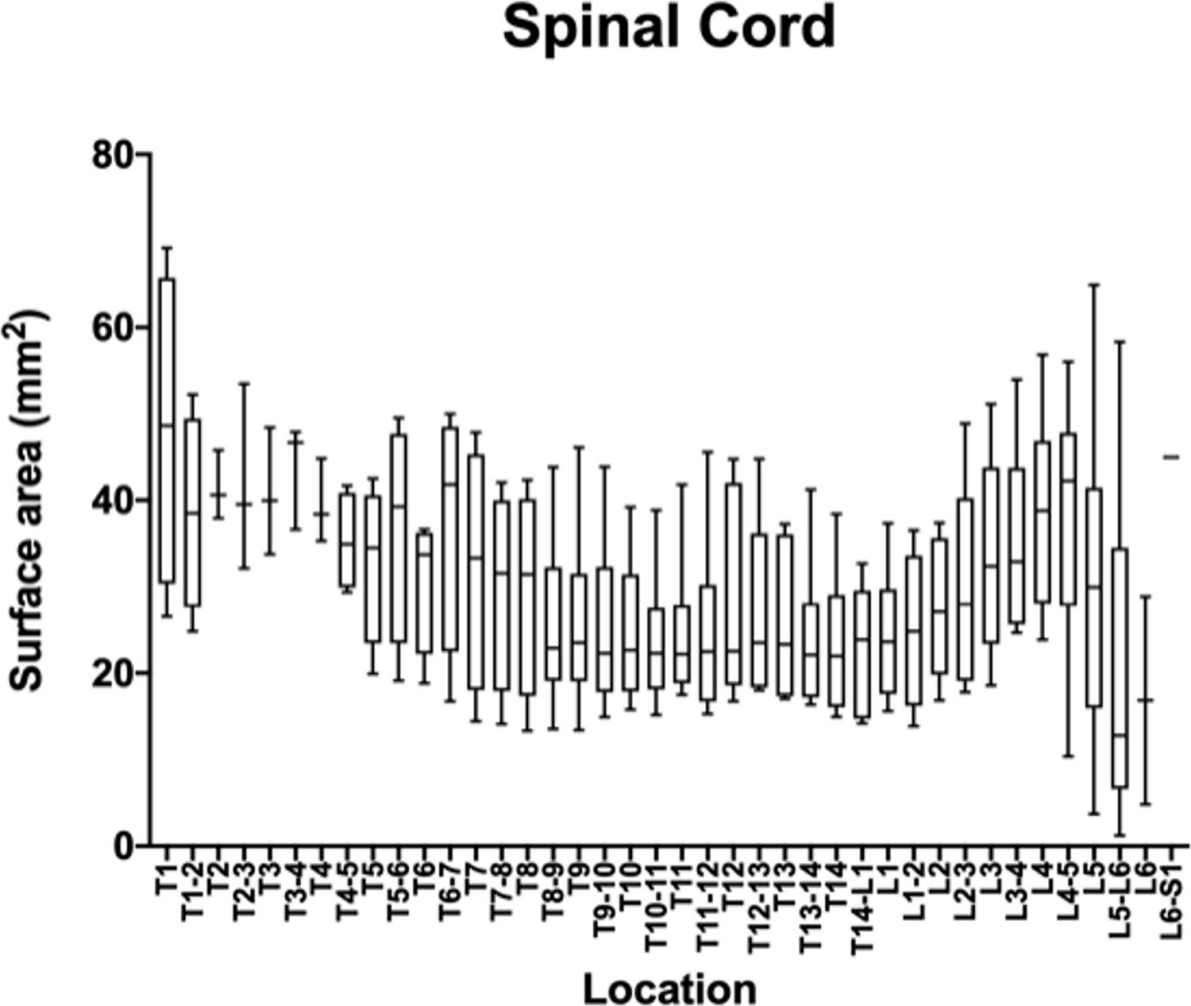
Graph of the spinal cord surface area (mm2) in 6 Yucatan sows. The median, 75^th^, 25^th^ percentile and the range are shown for each thoracolumbar vertebral segment.

**Fig 8:**
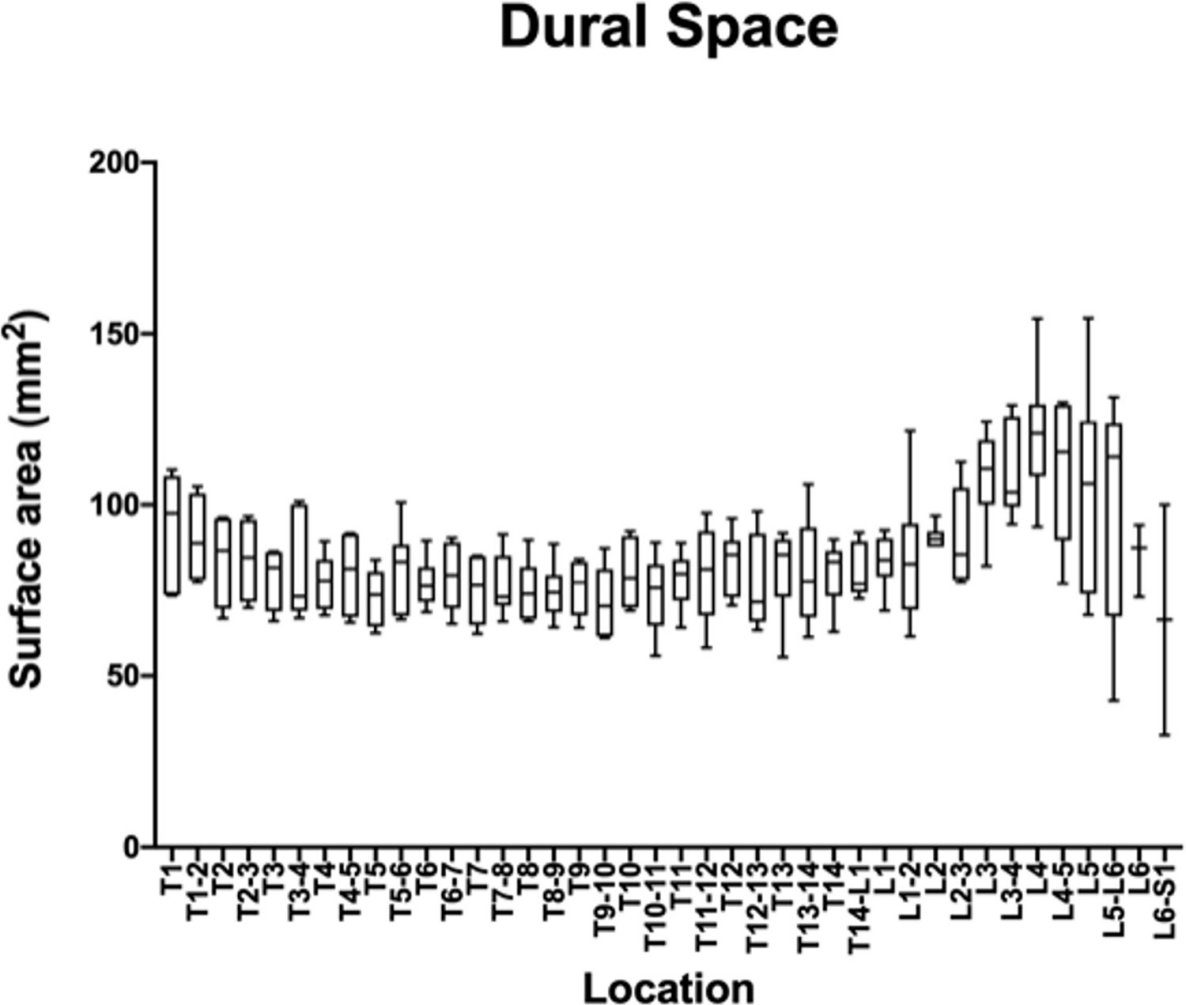
Graph of the dural space surface area (mm2) in 6 Yucatan sows. The median, 75^th^, 25^th^ percentile and the range are shown for each thoracolumbar vertebral segment.

**Fig 9:**
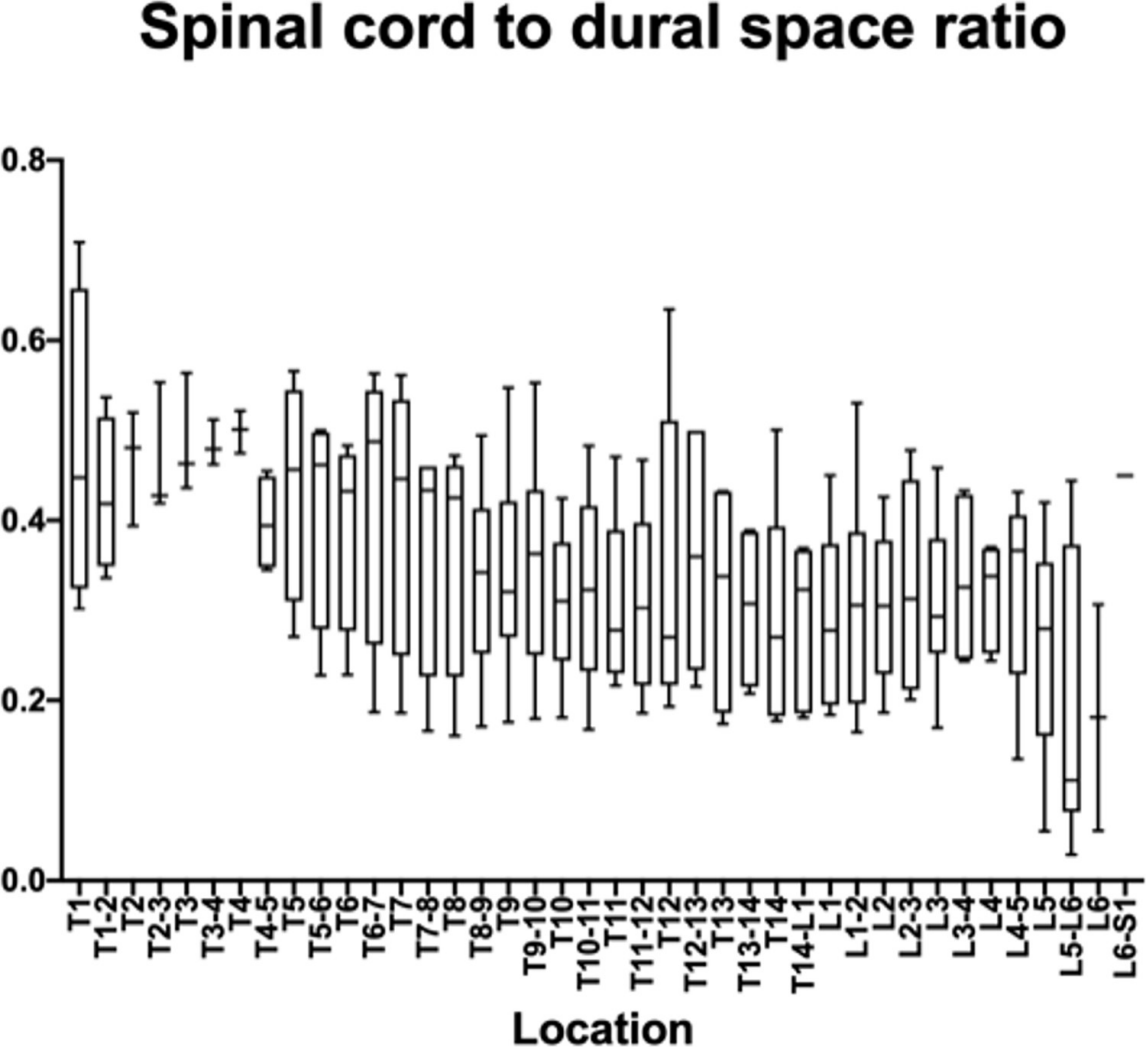
Graph of the spinal cord to dural space ratio in 6 Yucatan sows. The median, 75^th^, 25^th^ percentile and the range are shown for each thoracolumbar vertebral segment.

**Fig 10:**
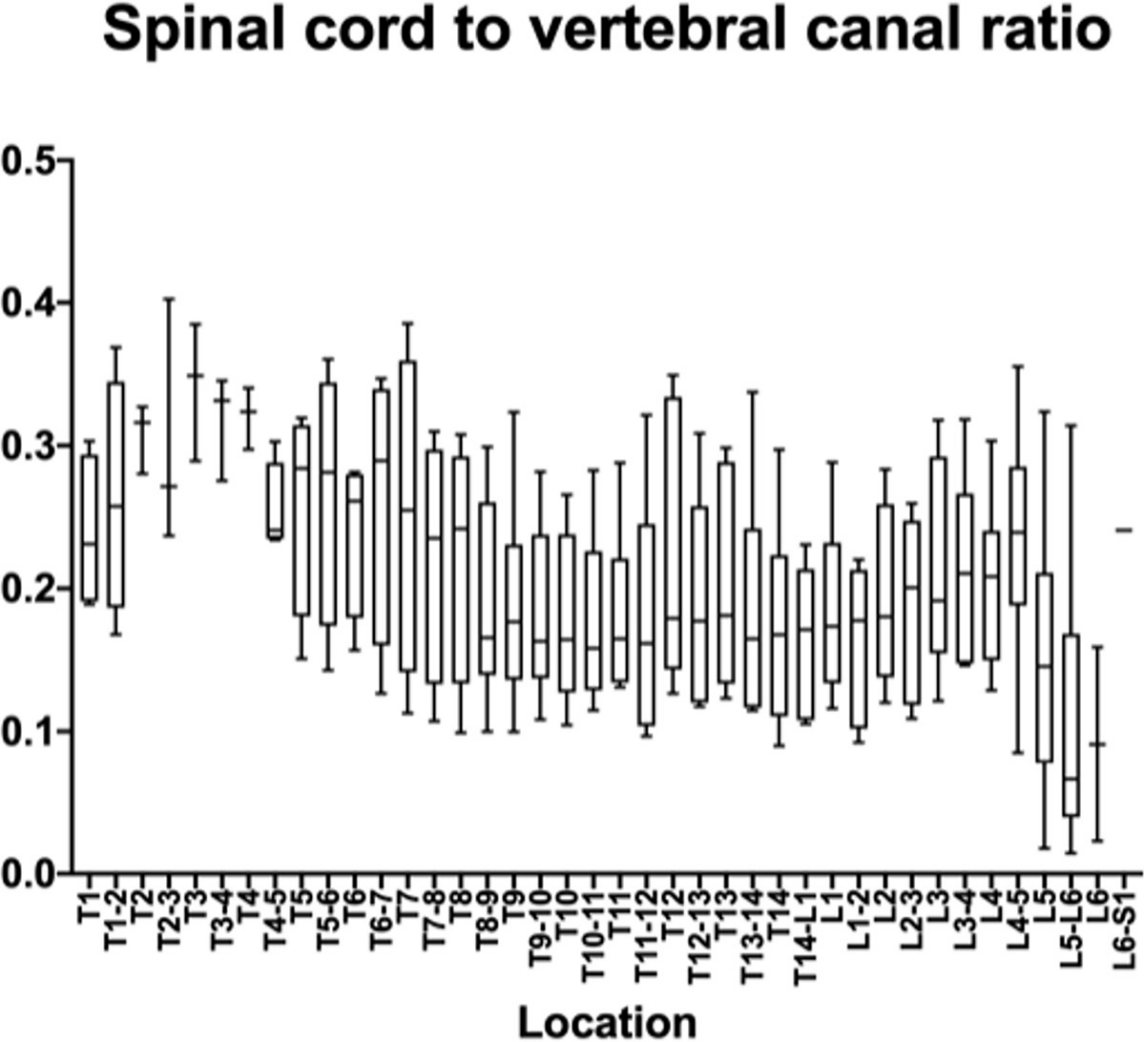
Graph of the spinal cord to vertebral canal ratio in 6 Yucatan sows. The median, 75^th^, 25^th^ percentile and the range are shown for each thoracolumbar vertebral segment.

**Fig 11:**
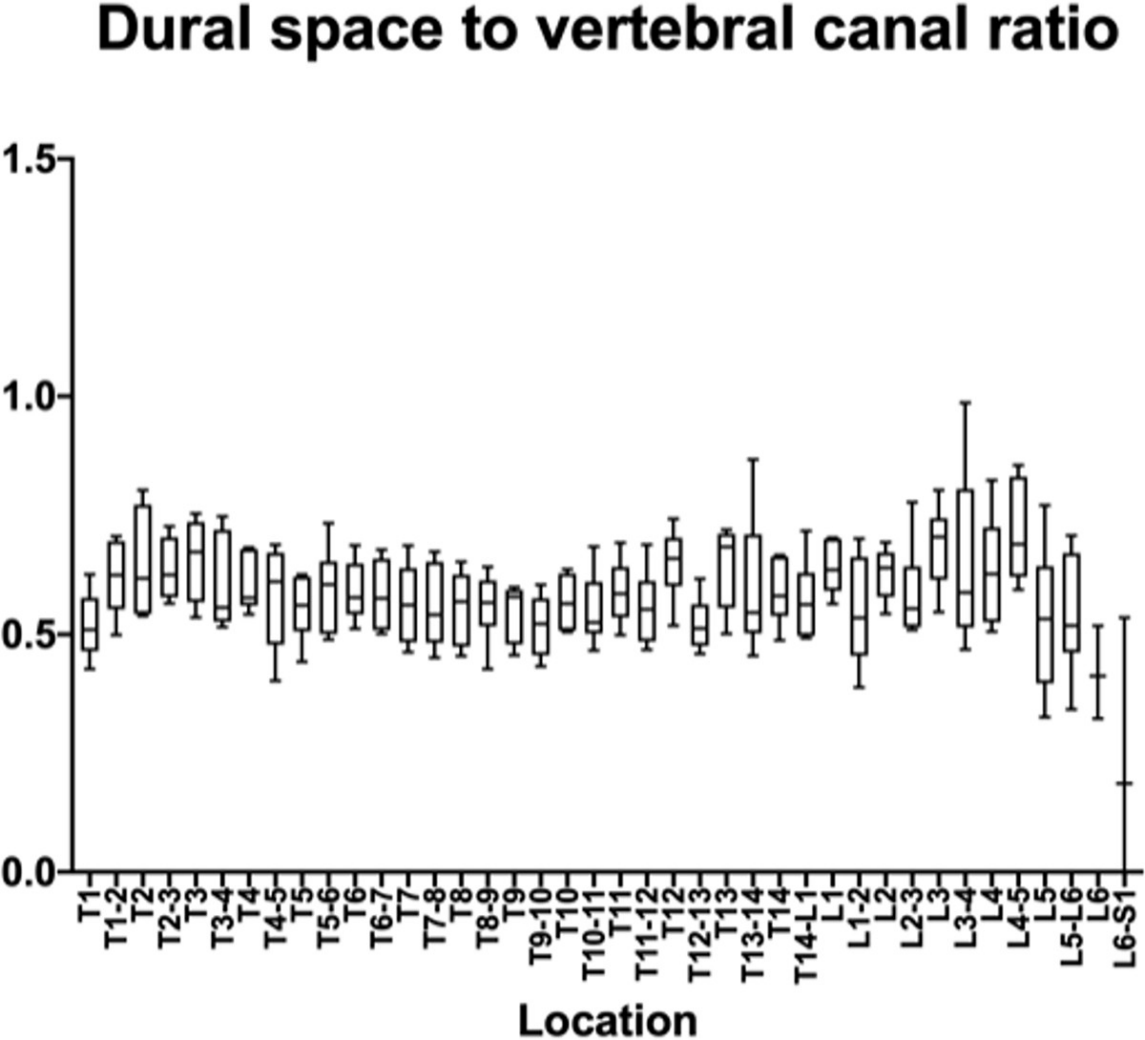
Graph of the dural space to vertebral canal ratio in 6 Yucatan sows. The median, 75^th^, 25^th^ percentile and the range are shown for each thoracolumbar vertebral segment.

The measurements and ratios for 232 vertebral segments out of 240 were normally distributed. Figs 6–11 show the morphometric outcomes at each spinal segment. The median, 75^th^, 25^th^ percentile, range are shown with a box and whisker plot for each thoracolumbar vertebral segment. The coefficient of variation (CV) for the vertebral canal surface area and dural space (DS) was significantly lower (p<0.0001) compared to that of the spinal cord (SC) and independent of the vertebral segment. The CV for the DS:VC ratio was significantly lower (p<0.0001) than the SC:VC and SC:DS ratios and was independent of the vertebral segment. The CV of the spinal cord surface area, the SC:DS and the SC:VC ratios were large compared to the smaller values for the VC and DS areas and the DS:VC ratios (p < 0.0001).

A significant correlation between T1-S1vertebral spine length and body weight was not shown (p = 0.0507) although the correlation coefficient, 0.81 showed a positive trend. Three sites (L5-6, T13-14 and L3-4) showed strong correlations (r = -0.84, -0.93, -0,88 respectively) between VC and DS surface area. No correlation could be shown for the SC surface area. The three ratios randomly showed strong correlation at three sites (T3, T2-3 and T12).

## Discussion

The CT-guided myelography technique described here provides a consistent method of examining the thoracolumbar spine in Yucatan swine. CTM was found to be a non-invasive and safe procedure. CT-guidance of spinal needle placement is important due to the difficulty of locating osseous landmarks like dorsal spinous processes due to large amounts of subcutaneous tissues and thick skin in the swine in this study population. This study also provides reference values in healthy Yucatan pigs for spinal CT morphometry that may be useful for future SCI patients and studies.

An important aspect of this technique was the placement in sternal recumbency in order to maintain the position of the sow and spine in the pre- and post-contrast scans. Spinal CT in dogs is most commonly performed in dorsal recumbency, however, this position does not allow access for spinal needle placement and dogs are repositioned in lateral recumbency for placement of the spinal needle to perform CTM. Dogs then have to be repositioned in dorsal recumbency and the pre- and post-contrast scans do not have identical positioning for direct comparison. Fluoroscopy can also be used for image guided spinal needle placement requires movement among imaging modalities between pre- and post-contrast scans, again resulting in changes in position. Performing CT-guided injection and scan all in the same position obviates the need to reposition for image acquisition, and pre- and post-contrast scans are aligned precisely for side-by-side anatomical assessment in an image viewer.

Variability in swine lumbar anatomy has been reported and is consistent with the variable number of lumbar vertebral segments in this study [5]. While all sows had 14 thoracic vertebrae, three had 5 and three had 6 lumbar vertebrae. Of the quantitative measurements, the spinal cord surface area had the widest range of values and the greatest coefficient of variance (CV) while those parameters for the vertebral canal had a low CV. Comparatively, of the morphometric ratios, the DS:VC, had the lowest CV while the spinal cord ratios to DS and VC had the highest (>30). This may be due to variation in manual delineation of the spinal cord margins and variable subarachnoid space filling among vertebral sites. This is supported by the smaller standard deviation and CV for the vertebral canal surface area which does not rely on contrast filling for margin identification. The shape and degree of the contrast filling of the subarachnoid space was variable and it was considered that the hand drawing of the spinal cord and the dural space surface areas would be more variable due to that. Blooming artifact at the margin between the inner border of the contrast column and the spinal cord, even using the same viewing windows and levels, could create variability. This would also be the case for the measurement of the dural space surface area.

Based on the study results, the DS:VC ratio may be the best measure for porcine SCI models. However, given the large coefficients of variation and standard deviations in surface area measurements for the spinal cord and dural space found in this study, intra-animal measures are probably warranted. Within the size range of sows in this study, correlations between spinal morphometry and body weight and spinal length were not shown. In a previous study of CTM morphometry in the cervical and lumbar segments of miniature swine, the spinal cord and vertebral canal surface areas were compared to the vertebral body surface area at different ages to assess for differences from skeletal immaturity to maturity [17]. The current study in Yucatan swine establishes a method to quantify spinal cord and dural space ratios and provides reference values for them. In a study using CTM morphometry to diagnose degenerative myelopathy in dogs, it was found that SC:DS ratios were smaller for dogs with degenerative myelopathy compared to healthy controls [11]. Reference ranges, standard deviation and coefficient of variance were not calculated in that study population. However, the work suggests that CTM can be used to assess myelopathy compared to controls.

Limitations of this technique included poor filling of the subarachnoid space from the first to fifth thoracic vertebrae some sows. Although not done, the head can be elevated above the cranial thorax in order to pool the contrast medium in this location if required. Epidural contrast extravasation did occur but was minor and did not prevent visualization of the dural contrast. Due to the lack of superimposition in CT transverse images, the presence of epidural contrast did not hinder the morphometric assessment of the spinal cord, canal or dural space areas.

## Conclusion

CT myelography can be performed consistently in Yucatan sows in sternal recumbency using CT-guided spinal needle insertion into the ventral subarachnoid space at L3-4, and a dose of 0.3mg/kg fills the length of the thoracolumbar spine with one injection. Reference values for spinal morphology have a low coefficient of variation for the vertebral canal surface area and the dural space: vertebral canal ratio and may serve for comparison in future studies using this animal model.

## Supporting information

S1 Data (XLSX)
